# Analysis of factors affecting the postoperative drainage in patients with abdominoplasty with circumferential liposuction

**DOI:** 10.3389/fsurg.2025.1581931

**Published:** 2025-04-25

**Authors:** Ruomeng Yang, Zhen Song, Jian Wang, Hongwei Liang

**Affiliations:** ^1^ The Department of Plastic Surgery, The Fifth Clinical Medical College of Henan University of Chinese Medicine (Zhengzhou People’s Hospital), Zhengzhou, China; ^2^ The Department of Plastic Surgery, Henan Provincial People’s Hospital, Zhengzhou, China

**Keywords:** abdominoplasty, liposuction, drainage, volume analysis, lipoabdominoplasty

## Abstract

**Background:**

Abdominoplasty combined with circumferential liposuction serves as a reliable and indispensable method for aesthetic body contour. This study aims to investigate the factors that influence drainage volume and duration after the procedure.

**Methods:**

A retrospective study of 89 patients who received abdominoplasty with circumferential liposuction between January 2021 and October 2024 was conducted. Total drainage volume and duration were recorded. Univariable and multivariable linear regression and correlation analyses were utilized to identify the correlation between postoperative drainage and patient-related, and operation-related variables.

**Results:**

The mean total drainage volume was 420.6 ± 220.8 ml, and the mean drainage duration was 6.8 ± 1.9 days. Based on the univariate and multivariate linear regression analysis, several statistically significant associations were identified. BMI (*p* = 0.001), volume of lipoaspirate (*p* = 0.001), and weight of resected tissue (*p* < 0.001) were significantly associated with the drainage volume. BMI (*p* < 0.001), operation time (*p* < 0.001), volume of lipoaspirate (*p* < 0.001), and weight of resected tissue (*p* = 0.01) were risk factors influencing drainage duration.

**Conclusions:**

BMI, volume of lipoaspirate, and weight of resected tissue were associated with drainage volume and duration. Operation time was an independent factor influencing drainage duration.

## Introduction

Abdominoplasty has evolved significantly in plastic surgery since its introduction (1, 2). It is now a widespread and essential procedure aiming to restore the abdomen by excision of excess skin and fat, as well as the plication of the fascia (3). Diverse protocols employ tailored approaches based on skin redundancy severity and musculofascial integrity (4, 5). Liposuction was initially utilized with abdomen esthetic surgery to improve contour (6, 7). Since then, the concurrent application of abdominoplasty and liposuction have been widely performed and evolved as lipoabdominoplasty, which can produce a satisfactory aesthetic appearance (8). Although controversy existed regarding combined procedures due to flap perfusion risks (8), emerging evidence supports the safety of modified techniques undermining limited supraumbilical tunnel to preserve flap perfusion (9–11).

Nowadays, people have become increasingly demanding of their body contour, particularly hip-waist proportions. Abdominoplasty combined with circumferential liposuction can serve as a reliable and indispensable tool for achieving the desired objectives. Closed suction drainage remains standard for preventing seroma and dead space complications (12), though optimal drain duration remains debated (13, 14). Moreover, observation of drainage volume offers crucial information regarding the patient's postoperative recovery progress, while prolonged retention increases infection risks and impairs patient mobility and quality of life (15).

Current evidence on clinical factors affecting drainage volume after abdominoplasty remains limited (16). In this study, we measured the daily drainage volume in cases of abdominoplasty with circumferential liposuction and analyzed the factors that influence drainage volume and time of drain removal.

## Methods

### Study population and data resource

A retrospective study of 89 patients who received abdominoplasty with circumferential liposuction between January 2021 and October 2024 was conducted. The inclusion criteria were as follows: (1) abdominal skin and musculofascial laxity from postpartum status, massive weight loss (>15% TBW), or post-bariatric surgery; (2) age between 18 and 60; (3) documentation of BMI stability (<2 kg/m^2^ fluctuation) for ≥12 months. The exclusion criteria were as follows: (1) history of previous abdominoplasty; (2) chronic diseases including cardiovascular diseases, metabolic diseases, and immune diseases; (3) insufficiency of documentation regarding the records of surgery and postoperative drainage. Following approval granted by the Medical Ethics Committee of The Fifth Clinical Medical College of Henan University of Chinese Medicine (Zhengzhou People's Hospital). The study was carried out in adherence to the principles outlined in the Declaration of Helsinki. All participants provided their written informed consent before the initiation of the study.

The following data were obtained from the medical database: patient-related variables, including age, body mass index (BMI), smoking history, preoperative hemoglobin protein (Hb), preoperative activated partial thromboplastin time (APTT), preoperative thrombin time (TT); and operation-related variables, including operation time, blood loss, volume of tumescent fluid injected, volume of lipoaspirate, thickness of flap, weight of resected tissue; total drainage volume, drainage duration.

### Surgical technique

All the patients were performed under general anesthesia by the same surgeon at our hospital. Typically, the midline suprapubic incision was designed approximately 4–6 cm above the fourchette. The amount of redundant skin to be excised is determined by estimating suture tension through the pinch test. First, liposuction was performed in the abdomen and lower back. Then, two subcutaneous tunnels were created 5 cm lateral to the midline in the supraumbilical region through liposuction. The supra-scar zone should be designated as a liposuction-free area to preserve the vascular network of the abdominal flap and minimize risks of wound healing impairment. Subsequently, the marked skin and underlying subcutaneous tissue were resected, while the Scarpa fascia was preserved. The flap was dissected up to the xiphoid and costal margins. The plication of the rectus abdominis muscles and the aponeuroses of the external oblique muscles were performed. Then, the upper flap was pulled down and the incision was closed using layered suture technique. Two closed suction drains were placed, and all patients were retained in the hospital until the drains were removed.

In our study, the primary outcomes were drainage volume and drainage duration. The drainage volume was defined as the total volume of fluid collected from wound ooze, commencing from the day of surgery until the removal of both drains. The drains were removed when the output remained below 30 ml for consecutive two days.

### Data analysis and statistics

For continuous variables, we presented the median and range for non-normally distributed data, and the mean ± standard deviation (SD) for normally distributed data. Categorical variables were reported as counts and proportions. To assess the relationship between postoperative drainage and independent patient variables, we conducted a univariate linear regression analysis. Variables that demonstrated a significant univariate relationship (*p <* 0.05) were included in the subsequent multivariate linear regression analysis, with postoperative drainage as the dependent variable. Linear correlations between variables were evaluated using the Pearson correlation coefficient (Pearson's *r*) for normally distributed variables and Spearman's rank correlation coefficient (Spearman's rho) for non-normally distributed variables. All statistical analyses were conducted using SPSS version 29.0 (SPSS Inc., Chicago, IL). Variables with a *p* less than or equal to 0.05 were considered statistically significant. All *p*-values were two-tailed.

## Results

A total of 89 patients underwent abdominoplasty with circumferential liposuction surgery between January 2021 and October 2024. All patients were women, with a mean age of 34.5 ± 5.2 years and a mean BMI of 23.0 ± 2.1 kg/m^2^. Among the patients, 4.8% (*n* = 3) were smokers. All patients were devoid of comorbidities. Preoperatively, Hb, APTT, and TT had an average value of 128.1 ± 10.3 g/L, 26.4 ± 4.5 s, and 17.7 ± 3.6 s, respectively. During the operation, the mean operation time was 367.4 ± 77.2 min. The mean volume of tumescent fluid injected, and the mean volume of lipoaspirate was 2,488.9 ± 756.5 ml and 1,716.9 ± 571.8 ml. The mean thickness of flap was 3.5 ± 0.7 cm. The mean weight of resected tissue was 1,540.4 ± 557.6 g, and the mean blood loss was 60.5 ± 19.2 ml. Overall, the average total drainage volume was 420.6 ± 220.8 ml, and the mean time of drainage was 6.8 ± 1.9 days. Patients' information was presented in Supplementary Table 1. Regarding postoperative complications, skin flap necrosis was observed in 2 patients, and the wound healed after dress changing. Transfusion was used in 1 patient (1%). No seroma, hematoma and infection were seen in any patients.

Univariable and multivariable linear regression analyses were employed to identify the factors influencing the total drainage volume (Supplementary Table 2). In the univariable analysis, BMI (*p* < 0.001), preoperative APTT (*p* < 0.001), operation time (*p* < 0.001), volume of tumescent fluid injected (*p* < 0.001), volume of lipoaspirate (*p* < 0.001), blood loss (*p* < 0.001), and weight of resected tissue (*p* < 0.001) showed significant associations with the total drainage volume. In the multivariable analysis, only BMI (*p* = 0.001), volume of lipoaspirate (*p* = 0.001), and weight of resected tissue (*p* < 0.001) were significantly associated with the drainage volume.

The factors influencing the time of drainage output were identified through univariate and multivariate linear regression analyses (Supplementary Table 3). Among the factors, BMI (*p* < 0.001), preoperative APTT (*p* < 0.001), operation time (*p* < 0.001), volume of tumescent fluid injected (*p* < 0.001), volume of lipoaspirate (*p* < 0.001), blood loss (*p* < 0.001), and weight of resected tissue (*p* < 0.001) showed significant associations with the drainage duration through univariate analysis. However, BMI (*p* < 0.001), operation time (*p* < 0.001), volume of lipoaspirate (*p* < 0.001), and weight of resected tissue (*p* = 0.01) were significant factors of drainage duration in the multivariate analyses. For multivariable linear regression analyses, all VIF values remained <5 except for volume of lipoaspirate (VIF = 6.4) and volume of tumescent fluid injected (VIF = 5.9).

The linear correlation between the analyzed variables except for drainage volume and duration was assessed using Pearson correlation coefficient and Spearman's rank correlation (Supplementary Table 4). BMI, preoperative APTT significantly influenced the volume of tumescent fluid injected, volume of lipoaspirate, and weight of resected tissue. While APTT also influence operation time and blood loss. Blood loss and wight of resected tissue were also impacted by volume of tumescent fluid injected and lipoaspirate.

## Discussion

Concurrent abdominoplasty and extensive liposuction have become a standard treatment for abdominal contour deformities caused by weight fluctuations, aging, or pregnancy (3). Contemporary evidence confirms the safety and efficacy when combining the two procedures (3, 8). To avoid the occurrence of seroma and hematoma, closed suction drains are routinely placed (17). Seroma refers to serous fluid accumulation, typically caused by postoperative tissue exudation. Hematoma, in contrast, results from vascular injury with blood pooling in tissue spaces, often occurring immediately or within hours postoperatively. In abdominoplasty, both conditions may prolong drainage duration but require distinct management approaches. Seroma relies on drainage optimization, whereas hematoma necessitates exclusion of active bleeding. Nevertheless, prolonged drainage increases infection risks, patient discomfort, and healthcare costs (18–20). Through univariable and multivariable linear regression analyses, patient- and operation-related factors were identified, to appropriately manage care of the patients. For multivariable linear regression analyses, the elevated VIF between volume of lipoaspirate (VIF = 6.4) and volume of tumescent fluid injected (VIF = 5.9) reflected their interdependence of the procedure of liposuction. Despite statistical collinearity, both were retained due to distinct clinical interpretations: tumescent fluid remaining in tissue gaps might be drained out postoperatively, whereas volume of lipoaspirate directly related to tissue trauma and causing an increase in reactive exudation in tissue.

Numerous studies have investigated factors affecting postoperative drainage volume and duration across various surgical specialties, including breast surgery, nephrectomy, and gastrointestinal or osteoarticular procedures (19–22). While extensive abdominoplasty research has focused on postoperative seroma formation and risk factors, limited evidence exists regarding drainage characteristic analysis. Our findings demonstrate that both patient demographics and surgical variables significantly influenced postoperative drainage patterns, consistent with previous reports (14, 20). Bonnema et al. (23) demonstrated that drainage fluid composition resembles peripheral lymphatic fluid, containing cellular components with higher protein content and absent fibrinogen. This suggests that the gradual reduction in drainage volume reflects the natural wound healing process.

The drainage duration varies across different types of surgery. For instance, the mean period was 3.52 ± 0.71 days in laparoscopic nephrectomy surgery (19), while the median duration was 12 days in tissue expander breast reconstruction (14). In our study, mean drainage duration was 6.8 ± 1.9 days with 420.6 ± 220.8 ml total output. While output typically declined progressively, intermittent fluctuations occurred due to kinking of the drain tubes, change in position, and activity increase. Thus, to avoid overhasty removal of drains, the criterion for drains removal was a daily output less than 30 ml for two consecutive days and it has been reported as a popular protocol (24, 25).

While prior studies report positive correlations between age and drainage duration and volume (13, 26), our analysis revealed no significant associations. The wound-healing response involves a complex interplay of mechanisms, including the sequential phases of hemostasis, inflammation, proliferation, and remodeling (13). Additionally, it is widely acknowledged that aging skin wounds heal in a manner distinct from younger wounds, characterized by delayed closure rates and mechanically weaker tissues (26). Age-related wound healing alterations, including delayed cellular proliferation, reduced fibrosis, and impaired extracellular matrix remodeling, theoretically predispose older patients to prolonged drainage. Our study revealed that the mean age of all female patients was 34.5 ± 5.2 years, spanning a range from 25 to 52 years. However, differing from previous reports, our findings indicate that there is no significant association between age and drainage duration and volume. Hence, increasing the study sample may yield different outcomes regarding the correlation between age and postoperative drainage.

As reported by other researchers, patients with higher BMI exhibited a significantly prolonged drainage duration and drainage volume compared to those with a lower BMI (18, 20, 27). Canbek et al. (17) further proposed that patients with a BMI of 40 or higher had longer periods of drainage. In the present study, the mean BMI was 23.0 ± 2.1 kg/m^2^, ranging from 18.5 kg/m^2^ to 30.6 kg/m^2^. We also found that BMI had a significantly positive association with both drainage duration and drainage volume. Patients with higher BMI typically exhibit greater abdominal skin laxity and thicker subcutaneous adipose layers. To achieve optimal aesthetic outcomes, they generally require more extensive liposuction and tissue excision during surgery. Additionally, we observed a correlation between operation time and drainage duration. The weight of the resected tissue appeared to be a risk factor for prolonging drainage time and increasing the total drainage volume. Prior studies have identified mastectomy weight as a factor influencing the volume of drainage output (13).

Our analysis identified lipoaspirate volume as a key risk factor of prolonged drainage duration and increased output. Liposuction-related blood loss remains a critical concern. The advent of the “tumescent technique”, which involves the subcutaneous infiltration of a large volume of vasoconstrictive solution, has led to a substantial reduction in blood loss, with aspirate containing approximately one percent of blood (28). Current evidence reveals 12.4 ml whole blood loss per 1,000 ml aspirate (29), aligning with our findings that individual variations in aspirate volume directly influence hemorrhagic exudate levels.

Postoperative drainage fluid comprises hemorrhagic and serous exudate. Hemorrhage control represents only one aspect of drainage management. In all cases, we preserved Scarpa fascia and utilized Progressive Tension Sutures (PTS). It has been proved that preservation of Scarpa fascia and PTS have been demonstrated to be effective in preventing dead space and seroma formation (30–32). Our study has several clinical implications. First, establishing key influencing factors may reduce reliance on subjective experience for drain removal. Second, identifying high-risk patients allows preemptive interventions to minimize complications, such as seroma or infection. Third, Dynamic drainage monitoring may guide compression therapy and activity restrictions for diverse patients to enhance recovery. Finally, shortening unnecessary drain retention could reduce hospitalization costs and healthcare burdens.

There exist limitations to our study. Firstly, the sample size was relatively small, and the study's reliance on a single-center dataset introduce potential selection bias, the majority of patients were from a normal population with relatively low BMI, which limited the conclusion to this specific population and precluded further subgroup analysis of the samples. Secondly, this study fails to evaluate long-term aesthetic outcomes and postoperative complications, thereby restricting comprehensive evaluation of the surgery in the long term., Additionally, potential risk factors such as diabetes, hypertension, smoking et al. could not be assessed due to their low incidence within the study population. Furthermore, elucidating the correlation between complications, such as seroma, and these factors is challenging due to the small number of complications occurrence in this study. In subsequent research, the sample size will be expanded, and subgroups will be established to enhance the statistical power of our analysis and strengthen the clinical relevance of our conclusions.

## Conclusions

In the procedure of abdominoplasty with circumferential liposuction, BMI, volume of lipoaspirate, and weight of resected tissue were factors affecting the total drainage volume. BMI, operation time, volume of lipoaspirate, and weight of resected tissue were significantly associated with drainage duration. While further investigations are warranted, these findings provide plastic surgeons with evidence-based insights to optimize perioperative drainage protocols tailored to individual patient characteristics.

## Data Availability

The raw data supporting the conclusions of this article will be made available by the authors, without undue reservation.
