# Supplementary material for: Analysis of factors affecting the postoperative drainage in patients with abdominoplasty with circumferential liposuction
Source: Front Surg. 2025 Apr 25;12:1581931. doi: 10.3389/fsurg.2025.1581931 (PMC12062131; doi:10.3389/fsurg.2025.1581931)
Supplement: Supplementary file 1 [file Table1.docx]

**TABLE 1 Demographic and clinical characteristics of patients undergoing abdominoplasty with circumferential liposuction (n = 89)**

| **Variable** | **Mean ± SD (range)** | **Value (%)** |
| --- | --- | --- |
| Patient-related characteristics |  |  |
| Age (years) | 34.5 ± 5.2 |  |
| BMI (kg/m^2^) | 23.0 ± 2.1 |  |
| Smoking history |  | 3 (4.8) |
| Preoperative Hb (g/L) | 128.1 ± 10.3 |  |
| Preoperative APTT (s) | 26.4 ± 4.5 |  |
| Preoperative TT (s) | 17.7 ± 3.6 |  |
| Operation-related characteristics |  |  |
| Operation time (min) | 367.4 ± 77.2 |  |
| Volume of tumescent fluid injected (mL) | 2488.9 ± 756.5 |  |
| Volume of lipoaspirate (mL) | 1716.9 ± 571.8 |  |
| Thickness of flap (cm) | 3.5 ± 0.7 |  |
| Weight of resected tissue (g) | 1540.4 ± 557.6 |  |
| Blood loss during operation (mL) | 60.5 ± 19.2 |  |
| Total drainage volume (mL) | 420.6 ± 220.8 |  |
| Duration of drainage (day) | 6.8 ± 1.9 |  |
| BMI, body mass index; Hb, hemoglobin; APTT, activated partial thromboplastin time; TT, thrombin time | | |
