# Supplementary material for: Analysis of factors affecting the postoperative drainage in patients with abdominoplasty with circumferential liposuction
Source: Front Surg. 2025 Apr 25;12:1581931. doi: 10.3389/fsurg.2025.1581931 (PMC12062131; doi:10.3389/fsurg.2025.1581931)
Supplement: Supplementary file 2 [file Table2.docx]

**TABLE 2 Univariable and multivariable linear regression analyses of factors influencing the total volume of drainage (n = 89)**

|  | Univariable |  |  | Multivariable |  |  |
| --- | --- | --- | --- | --- | --- | --- |
|  | *β* | SE | Value of *p* | *β* | SE | Value of *p* |
| Age | -2.18 | 6.390 | 0.743 | - | - | - |
| BMI | 59.774 | 11.282 | <0.001 | 23.217 | 7.322 | 0.001 |
| Smoking history | 107.992 | 123.416 | 0.387 | - | - | - |
| Preoperative Hb | 6.363 | 3.530 | 0.079 | - | - | - |
| Preoperative APTT | -29.478 | 7.143 | <0.001 | -5.885 | 3.856 | 0.133 |
| Preoperative TT | -17.356 | 10.8 | 0.116 | - | - | - |
| Operation time | 2.110 | 0.247 | <0.001 | 0.241 | 0.240 | 1.066 |
| Volume of tumescent fluid injected | 0.221 | 0.023 | <0.001 | 0.002 | 0.033 | 0.960 |
| Volume of lipoaspirate | 0.332 | 0.025 | <0.001 | 0.146 | 0.045 | 0.002 |
| Blood loss | 7.240 | 1.145 | <0.001 | 1.271 | 0.948 | 0.186 |
| Thickness of flap | 69.792 | 52.157 | 0.212 | - | - | - |
| Weight of resected tissue | 0.333 | 0.027 | <0.001 | 0.126 | 0.035 | <0.001 |

BMI, body mass index; Hb, hemoglobin; APTT, activated partial thromboplastin time; TT, thrombin time
