# Supplementary material for: Analysis of factors affecting the postoperative drainage in patients with abdominoplasty with circumferential liposuction
Source: Front Surg. 2025 Apr 25;12:1581931. doi: 10.3389/fsurg.2025.1581931 (PMC12062131; doi:10.3389/fsurg.2025.1581931)
Supplement: Supplementary file 3 [file Table3.docx]

**TABLE 3 Univariable and multivariable linear regression analyses of factors influencing drainage duration (n = 89)**

|  | Univariable |  |  | Multivariable |  |  |
| --- | --- | --- | --- | --- | --- | --- |
|  | *β* | SE | Value of *p* | *β* | SE | Value of *p* |
| Age | 0.015 | 0.031 | 0.635 |  |  |  |
| BMI | 0.590 | 0.097 | <0.001 | 0.284 | 0.053 | <0.001 |
| Smoking history | 0.694 | 0.602 | 0.256 |  |  |  |
| Preoperative Hb | 0.221 | 0.002 | 0.635 |  |  |  |
| Preoperative APTT | -0.286 | 0.063 | <0.001 | -0.055 | 0.028 | 0.054 |
| Preoperative TT | -0.025 | 0.055 | 0.652 |  |  |  |
| Operation time | 0.021 | 0.002 | <0.001 | 0.009 | 0.002 | <0.001 |
| Volume of tumescent fluid injected | 0.001 | 0.000 | <0.001 | 0.000 | 0.000 | 0.530 |
| Volume of lipoaspirate | 0.003 | 0.000 | <0.001 | 0.001 | 0.000 | <0.001 |
| Blood loss | 0.058 | 0.011 | <0.001 | -0.002 | 0.007 | 0.817 |
| Thickness of flap | 0.404 | 0.253 | 0.119 |  |  |  |
| Weight of resected tissue | 0.003 | 0.000 | <0.001 | 0.001 | 0.000 | 0.01 |

BMI, body mass index; Hb, hemoglobin; APTT, activated partial thromboplastin time; TT, thrombin time
