# Supplementary material for: Analysis of factors affecting the postoperative drainage in patients with abdominoplasty with circumferential liposuction
Source: Front Surg. 2025 Apr 25;12:1581931. doi: 10.3389/fsurg.2025.1581931 (PMC12062131; doi:10.3389/fsurg.2025.1581931)
Supplement: Supplementary file 4 [file Table4.docx]

**TABLE 4 Pearson correlation and Spearman’s rank correlation analyses between other variables.**

|  | Age | BMI | Preoperative APTT | Operation time | Volume of tumescent fluid injected | Aspirated volume | Blood loss | Flap thickness | Weight of resected tissue |
| --- | --- | --- | --- | --- | --- | --- | --- | --- | --- |
| Age | 1 | - | - | - | - | - | - | - | - |
| BMI | 0.576 | 1 | - | - | - | - | - | - | - |
| Preoperative APTT | 0.244 | 0.349 | 1 | - | - | - | - | - | - |
| Operation time | 0.158 | 0.106 | <0.001 | 1 | - | - | - | - | - |
| Volume of tumescent fluid injected | 0.396 | <0.001 | 0.012 | <0.001 | 1 | - | - | - | - |
| Volume of lipoaspirate | 0.173 | 0.001 | <0.001 | <0.001 | <0.001 | 1 | - | - | - |
| Blood loss | 0.312 | 0.689 | <0.001 | <0.001 | <0.001 | <0.001 | 1 | - | - |
| Flap thickness | 0.580 | 0.500 | 0.227 | 0.809 | 0.746 | 0.479 | 0.401 | 1 | - |
| Weight of resected tissue | 0.655 | 0.041 | 0.002 | <0.001 | <0.001 | <0.001 | <0.001 | 0.872 | 1 |

BMI, body mass index; APTT, activated partial thromboplastin time
